# Supplementary material for: Are carbon emissions trading and green financial instruments synergistic? -Comprehensive quantitative research based on content analysis
Source: PLoS One. 2024 Mar 7;19(3):e0298601. doi: 10.1371/journal.pone.0298601 (PMC10919601; doi:10.1371/journal.pone.0298601)
Supplement: S1 Data — (ZIP) [file pone.0298601.s001.zip › Data/地级市Z_F工作报告.zip!/╟α║ú/╬≈─■╩╨/└┤╫╘╣1⁄2╓┌║┼í╛┬φ┐╦╩2╛▌═°í┐_2016.docx]

各位代表：

现在，我代表市人民政府，向大会报告政府工作，请予审议，并请市政协委员和其他列席人员提出意见。

一、“十二五”圆满收官，西宁各项事业有了新突破

“十二五”时期是极不平凡的五年，是西宁发展势头好、城乡面貌变化大、人民群众得实惠多的五年。面对错综复杂的宏观环境和繁重艰巨的改革发展稳定任务，我们在省委省政府和市委的坚强领导下，紧紧围绕省委“三区”战略和市委建设人民满意的“生活之城、幸福之城”目标，经受住各种考验，付出艰辛努力，圆满完成了“十二五”规划确定的主要目标任务，经济社会发展取得重大成就，书写了无愧于时代的辉煌篇章。

这五年，我们始终坚持第一要务不动摇，持续保持快速发展，经济总量迈上大台阶。全社会固定资产投资、农民人均纯收入、地方公共财政预算收入指标提前一年完成“十二五”规划目标，地区生产总值和全社会固定资产投资跨过双千亿，主要经济指标增速位居省会城市前列。地区生产总值年均增长13.7%，分别高于全国全省5.9和2.9个百分点，是2010年的1.8倍。人均生产总值达到4.92万元，接近全国平均水平。五年累计完成固定资产投资4626亿元，是“十一五”的3.7倍。城乡居民人均收入年均分别增长10.8%和14.9%，高于全国0.1和2.2个百分点。社会消费品零售总额翻番，人均消费突破两万元。地方公共财政收入和预算支出翻番，全市总财力突破300亿元，达到326.8亿元，年均增长23.7%。经济总量的扩大，夯实了“生活之城、幸福之城”建设的基础。

这五年，我们着力提高发展质量和效益，传统产业与新兴产业双轮驱动、双向发力，现代产业体系初步形成。三次产业结构由2010年的3.9∶50.1∶46.0调整为3.3∶48.0∶48.7，实现由“二三一”向“三二一”的转变，构筑了一二三产协调发展的新格局。新型工业体系基本形成。坚持循环经济的发展方向，把转型升级放在更加突出的位置，工业在困难中保持年均17.4%的增速，支撑了全市经济平稳较快增长。加快传统产业提质升级，有色金属就地加工转化率大幅提升，“世界藏毯之都”初具规模，特色生物产业日益壮大。新兴产业见事早方向准，光伏全产业链体系基本形成；一批重大锂电产业项目建成投产，为打造千亿元锂电产业基地奠定坚实基础。突破多晶硅冷氢化等一批关键技术，开发区各企业拥有专利超过500余件，部分技术应用和单体技术装备跻身国内甚至国际先进行列。八大产业集群纵向延伸、横向耦合的现代工业体系基本形成。现代服务业成果丰硕。“夏都西宁”品牌内涵不断丰富，塔尔寺荣膺5A级景区，高原旅游集散功能快速提升，旅游接待人数和总收入分别是“十一五”末的1.8倍和3.4倍。引进浦发银行、广发证券等62家金融机构，金融业增加值年均增长18.1%，占生产总值比重达到10.8%，成为我市支柱产业之一。电子商务加速发展，农村通宽带率达到90%以上。朝阳物流中心等现代物流枢纽建成投用，新华联、万达综合体等大型高端商业业态引领发展新趋势，新兴服务业蓬勃发展。都市现代农业示范引领。油料、蔬菜等优势作物种植比重稳定在75%以上，培育合作社、家庭农牧场、种养大户2993家，全市龙头企业达到204家，农村土地流转率20.1%。农业增加值年均增速连续保持在5%以上，农畜产品加工转化率由45%提高到55%，夯实了高原绿色有机品牌农业发展基础。

这五年，我们不断推动园区、城区、县域齐头并进，整体统筹谋划能力不断提升，城乡区域协调发展取得新成效。树立协调发展理念,下好园区、城区、县域发展“一盘棋”，助推了全市经济社会持续健康发展。园区支撑作用不断增强。开发区一区多园、优势互补、错位发展的格局基本形成，承载高端产业、集聚人才技术、极具倍增潜力的五大基地正在崛起，工业总产值迈上千亿元台阶，成为带动工业经济发展的主战场主引擎主力军。城区发展注入新活力。四区经济年均增长14.6%，总量是“十一五”末的1.94倍，服务功能不断完善，市民对城市的归属感和荣誉感日益增强。城东区依托火车站改造带动建国路商圈建设，成为西宁对外开放新地标；城中区借助南川片区综合开发和园区工业快速发展，城区经济取得新突破；城西区借力海湖新区，促进服务业高端要素加速聚集；城北区北川河综合治理取得重大进展，山水生态宜居区成效凸显。县域经济速质双升。立足县域实际，放大特色优势，三县经济总量年均增长达到13.2%。湟中生产总值超过150亿元，县域经济总量稳居全省第一，塔尔寺大景区和多巴新城建设迈出实质性步伐；大通综合实力不断增强，东部新城、北川省级高新技术产业园区加快建设；湟源着力打造现代畜牧业示范县和文化旅游名县，丹噶尔古城保护、大华工业园加快实施。

这五年，我们努力促进城市扩容提质升级，不断加强基础设施建设，综合承载能力显著提高。先后实施一批基础设施重大项目，城市空间优化拓展，内涵和品位提升引领蝶变。发挥规划统筹作用，城镇体系不断优化。坚持顶层设计、全域规划，建立了覆盖全域、市县一体、城乡统筹的城乡规划体系，城市总体规划修改获得国务院批准。城乡一体化步伐不断加快，建成128个“美丽乡村”，提前完成52.5万农业人口转户工作，城镇化率达到68.92%，“一核、三极、多节点”的城镇体系格局基本形成。城市建设规模空前。着力实施“中疏”战略，“双增双减”取得新进展。建国路综合商务区建设，打造了城市东部新门户；新宁广场片区提升改造，构建了城市文化新高地；海湖新区成为总部经济和文化创意产业等高端服务业集聚的投资热土，铸就了城市新区建设的新辉煌。城市骨架不断伸展，启动南川片区开发，打造北川生态水城，建设西川片区基础设施。五年累计拆迁面积1642.26万平方米，是前十年的1.62倍，建成区面积增加37平方公里，达到118平方公里，总规确定的“两个中心，八个片区”带状组团式城市结构已经基本形成，城市面貌发生了历史性变化。基础设施日臻完善。西过境、南绕城高速公路全线贯通，百公里绕城高速环线建成并免费通行，初步构建了“外环内网”交通大格局。新建果洛路等一批主干道路，新增市政道路78.77公里，人均市政道路面积达到7.8平方米。新开通公交线路32条，新购更新公交车1588辆，成功入选国家“公交都市”创建示范城市。新建污水处理厂11座，排水干网349公里，污水日处理能力达到34.5万吨。城市管理成效显著。餐厨垃圾处理“西宁模式”在全国领先，生活垃圾无害化处理率达到95.3%。城市管理“网格化”体系全面形成，环卫市场化改革取得突破，全市道路机械化清扫率由不足20%提高到49.1%。创模工作通过专家评审，保持了国家卫生城市的荣誉称号。创建全国文明城市工作掀起热潮，市民素质和城市文明程度明显提高。

这五年，我们扎实推进生态文明建设，生态环境质量持续改善，可持续发展能力不断提升。坚持不懈治山、治气、治水、治污，努力让全市人民享有更多蓝天白云、绿水青山。人居环境日益改善。实施“清水入城”工程，建成北川、海湖、宁湖三大湿地，打造了“三河六岸”绿色景观生态廊道。实施天保、“三北”等工程，建成北山美丽园，建成区绿地覆盖率达到40.5%，人均绿地面积达到12平方米。大南山绿色屏障工程荣获“中国人居环境范例奖”，在西北省会城市中率先建成“国家森林城市”，再添一张国家级名片。持续推进大气污染治理。主城区“煤改气”在全国北方城市率先清零，黄标车淘汰走在全国前列，空气质量优良率大幅提升，位居西北省会城市前列。水生态文明试点城市建设扎实推进。实施引大济湟、防洪及流域管理世行贷款等重大水利工程，湟水流域综合整治取得重大进展，国控小峡口出境断面水质稳定达标。节能减排扎实推进。停改关产能落后企业30家，淘汰落后产能73.1万吨，规上工业单位增加值能耗较“十一五”末累计下降36.86%，全社会能源消费总量增速控制在4.9%以内。

这五年，我们注重保障和改善民生，加快发展各项社会事业，广大市民的获得感显著提升。扶贫攻坚深入推进。组织实施455个村整村推进、96个村易地搬迁等项目，五年累计23.88万名群众脱贫。实施农村饮水安全工程，解决了26.3万群众饮水安全问题。就业水平显著提高。累计新增城镇就业15.04万人，城镇登记失业率控制在2.5%以内，应往届高校毕业生总体就业率达到87%，农村劳动力转移就业195.18万人次，创业带动就业工作受到国务院表彰，荣膺“全国创业先进城市”称号。社保体系不断完善。城乡居民基本医疗保险并轨运行，农村社会养老保险实现全覆盖，城乡居民基础养老金由60元提高到130元，城乡居民医保人均筹资标准由240元、154.3元均提高到550元。城乡特困低保家庭实现应保尽保。住房条件明显改善。新建住房1639万平方米，人均住房面积达到31平方米，较“十一五”末增加5.5平方米。累计完成城镇保障性住房、棚户区改造住房17.26万套，解决8.37万户低收入家庭住房困难问题。教育事业全面发展。五年累计投入教育经费229亿元，是“十一五”的2.72倍。实施校安工程、教育布局调整、标准化学校、全面薄改等重大项目649个，新建改建西宁五中等城乡学校298所，办学条件发生历史性变化。义务教育和高中阶段教育普及目标提前实现。职业教育办学水平全面提升，城市职业技术学院填补了我市高职教育的空白。文化事业蓬勃发展。健全完善四级公共文化服务网络体系，文化产业增加值年均增长29.78%，国家公共文化服务体系示范区创建取得阶段性成效，公共文化服务水平显著提升。医疗保障能力全面提升。新增医疗卫生机构327家，基本公共卫生服务人均费用由15元增加到45元。构筑了城乡居民常规报销、大病保险和医疗救助“三道保障线”，群众“看病难、看病贵”问题有效缓解。

各位代表，五年来，我们心系民生，坚持每年都办成一批惠民实事，民生投入占财政支出的80%以上，累计投入是“十一五”的3.4倍，人民生活水平显著提高，各项社会事业取得了显著成就！

这五年，我们注重深化改革扩大开放，不断拓宽视野、破解难题，发展动力活力不断增强。按照省委改革上走在前的要求，以改革红利弥补区位劣势，打造全省改革高地。全力落实中央和省上改革试点。医药卫生体制改革走在全省前列，生态文明和司法体制改革取得阶段性成果，农村土地承包经营权确权登记颁证等试点工作取得新进展。简政放权取得新突破。市级非行政许可审批事项提前一年清零，行政审批事项总精简率达到71.6%，成为西北省会城市中行政审批项目最少的城市。出台了“权责清单”。深入推进商事制度改革，五年新增市场主体6.5万户，是“十一五”末的1.8倍。深化投融资体制改革，探索推进PPP融资等多种方式。金融服务实体经济的作用更加明显。经济、社会、生态等领域改革稳步推进。不断提升对外开放水平。充分利用青洽会、清食展、城洽会等会展活动，引进招商引资项目845个，累计到位资金2518.6亿元，较“十一五”增加2.5倍。国际航空口岸投入运营，新开通国际航线4条，西宁机场旅客吞吐量突破400万人次。火车新站投用，西格复线、兰新二线通车运营，西宁进入高铁时代。加强对外交流合作，国际友好城市和友好交流地区达到25对，实现了由传统内陆型向现代开放型城市的重大转型。

同时，法治西宁、平安西宁建设扎实推进，民族团结进步先进区创建工作卓有成效，社会安全、生产安全、食品药品安全、校园安全等工作统筹推进，未发生重特大安全事故。双拥工作连续8年受到省级表彰，军地军民关系融洽和谐。

五年来，我们不断加强政府自身建设，深入开展党的群众路线教育实践活动和“三严三实”专题教育，法治政府建设深入推进，政府执行力公信力明显增强。认真开展“向人民汇报、请人民评议”活动，倾听人民呼声，接受人民监督，共办结人大代表、政协委员建议、提案1611件。气象、工青妇、残疾人、红十字等工作全面发展。全面完成玉树震后援建任务，做出了省会城市应有的贡献。

各位代表，过去五年，在大事急事难事接踵而至的情况下，我们每前进一步都付出了艰辛探索和努力，也深化了对市情特征和发展规律的认识。我们深切地感到，必须坚持把学习贯彻习近平总书记系列重要讲话精神作为基本遵循和根本指南，融会贯通、学以致用，坚定不移地把中国特色社会主义在西宁坚持好拓展好，确保西宁各项事业始终沿着正确方向前进；必须坚决贯彻落实省委“三区”战略不动摇，按照“发展上做贡献，改革上走在前，稳定上做支撑”的重要要求，始终以生态文明优先理念统领经济社会发展，坚持走绿色低碳循环发展道路，夯实民族团结进步根基，为全面建成小康社会打下坚实基础；必须坚持发展第一要务不动摇，尽最大可能争取国家和省上支持与自身努力相结合，创造性地落实国家和省上一系列政策措施，坚持“一张蓝图绘到底，一茬接着一茬干”，促进全市经济社会实现跨越发展；必须坚持不懈解放思想，深化改革开放不停步，激发经济社会发展内生动力活力，充分利用新理念新思路破解发展中的各类难题；必须坚持营造风清气正的政治生态，展现不甘人后、善作善成的精神风貌，不断激发各级干部和各族群众干事创业的热情，形成推动经济社会发展的强大合力。

刚刚过去的2015年，面对经济下行压力持续加大的严峻形势，我们迎难而上，主动适应经济发展新常态，全力以赴推动经济社会持续健康发展，全面完成了市十五届人大六次会议确定的目标任务。经济发展上，把稳增长摆在更加突出位置，全年实现生产总值1131.62亿元，增长10.9%，增幅位居全国省会城市第二，稳增长得到国务院表彰。地方公共财政预算收入94.8亿元，增长13%，超省定目标2个百分点。固定资产投资增长10.1%，其中市属固定资产投资增长18.2%，一批大项目新项目相继建成和开工。社会消费品零售总额达到461.9亿元，增长11.6%。城乡居民人均可支配收入分别增长10.2%、8.7%。居民消费价格指数涨幅2.5%，处于近年来较低水平。转型升级上，从调存量和优增量两方面持续推进结构调整，加快培育新动能，高新技术产业、装备工业增加值分别增长16.7%和23%，高于全市工业增速。第三产业增加值占比近年来首次超过第二产业。旅游人次和总收入分别增长11.3%和23.4%，电子商务交易额同比增长17.5%，贷款余额突破4000亿元大关。城乡建设和生态上，成功跻身第二批国家新型城镇化综合试点城市，启动多巴新城总规、南川文化旅游商贸会展功能区建设规划及城市绿芯森林公园规划编制，北川河综合治理核心区基本建成。启动“畅通西宁”三年攻坚计划，按照系统化解决、综合性施策的思路制定出台了六大行动方案。建成150公里城市绿道。持续推进大气污染和工业污染综合治理，全年空气质量优良率达77.6%。改革开放上，医药卫生等上级试点改革走在全省前列，简政放权成效显著，行政审批服务局正式运行。成功举办光彩事业丝路行暨2015城洽会,与浦东、青岛等市区签订战略合作协议，全年招商引资到位资金840亿元，同比提高15%。民生福祉上，6大类39项民生实事项目全面完成，民生支出占财政总支出的81.4%。城镇新增就业3.14万人，就业困难人员实现就业2582人，当年脱贫4万人。社保、住房、稳价、教育、卫生、文化等民生事业取得新进展，改革发展成果更多更公平惠及广大市民。

各位代表，过去五年，我们共同生活的这座城市发生了深刻变化，焕发出新的生机与活力。这些成绩的取得，是省委省政府坚强领导、特殊关怀的结果，是市委团结带领全市各族人民艰苦奋斗、顽强拼搏的结果，是全市各级干部苦干实干、默默奉献的结果，是市人大、市政协和社会各界大力支持的结果。在此，我代表市人民政府，向全市各族干部群众，向人大代表、政协委员、各民主党派、工商联、无党派人士和各人民团体，向人民解放军驻宁部队指战员、武警官兵、公安干警和驻宁企业，向所有关心、支持西宁建设和发展的同志们、朋友们，致以崇高的敬意和衷心的感谢！

在看到成绩的同时，我们也清醒地认识到西宁发展和政府工作中还存在一些矛盾和问题：发展基础仍然薄弱，不协调、不平衡问题较为突出，综合实力不强；产业结构层次不高，竞争力、创新能力不强，转型升级、提质增效的任务艰巨；交通拥堵、空气污染等“城市病”日益突出，城市建设管理任务依然繁重；政府效能、依法行政的能力和干部作风需要进一步加强。对此，我们将高度重视，努力加以解决。

二、科学谋划“十三五”，全面建成小康社会

根据《中共西宁市委关于制定国民经济和社会发展第十三个五年规划的建议》，市政府制定了《西宁市国民经济和社会发展第十三个五年规划纲要（草案）》，本次大会审查批准后，我们将认真组织实施。这里简要说明如下。

（一）编制《纲要(草案)》的主要考虑和基本框架

“十三五”时期是我市率先全面建成小康社会的决胜阶段，是实现转型升级、跨越发展的关键时期。《纲要（草案）》全面贯彻了中央和省、市委的重大决策部署，充分考虑了“十三五”时期我市经济社会发展的趋势和要求，重点把握了以下几个方面：一是贯彻落实好习近平总书记系列重要讲话精神。《纲要（草案）》从指导思想、基本原则、主要目标到重点任务，坚持把学习贯彻习近平总书记系列重要讲话这一主线贯穿始终。二是贯彻落实好中央和省、市委“十三五”规划建议。《纲要（草案）》全面贯彻落实中央提出的五大发展理念和省、市委提出的主要目标、重点任务、重大举措，确保中央和省、市委重大决策部署落地生根。三是贯彻落实好全面建成小康社会的任务。《纲要（草案）》紧扣在全省率先全面建成小康社会的任务，厘清工作任务和时间节点，聚焦突出问题，明确破解难题、补齐短板的措施。四是贯彻落实好战略性、针对性、指导性和可操作性相结合的要求。《纲要（草案）》注重明确目标，细化措施，项目支撑。

根据市委《建议》，《纲要（草案）》明确提出了“十三五”时期经济社会发展的指导思想和原则，强调要坚持民生为本，增进福祉；坚持做强西宁，服务全省；坚持统筹城乡，协调发展；坚持生态优先，“五化”同步；坚持深化改革，扩大开放；坚持党的领导，强化保障。

《纲要（草案）》分为三个部分共八章。其中导语和第一、第二章构成第一部分，主要对“十二五”时期经济社会发展做出总结和评价，分析“十三五”期间全面建成小康社会决胜阶段的总体形势，提出发展的指导思想、基本原则和主要目标。第三至第七章构成第二部分，分别对应中央创新、协调、绿色、开放、共享五大发展理念，就我市分别实施的重点任务和重大举措进行阐述和部署。第八章和结束语构成第三部分，强调坚持党的领导，强化规划执行力。号召全市各级党政组织、广大党员干部和各族群众改革创新，奋发进取，为全面建成小康社会不懈奋斗。

（二）“十三五”时期经济社会发展的奋斗目标

《纲要（草案）》提出的主要奋斗目标是：不断强化创新协调绿色开放共享的发展理念，全面落实省委“131”总体要求，实现“12315”总目标，开启“幸福西宁”建设新征程。“一个率先”，即在全省率先全面建成小康社会，是全市“十三五”时期最重要的大局。“两个转型升级”，即推动产业和城市转型升级，是西宁“十三五”发展的基本路径，产业转型升级和城市转型升级分别体现了“在转型中发展”和“在发展中转型”的思路，两者共同构成了西宁实现全面小康目标的两大支柱。“三个示范基地”，即建设全省生态文明先行区、循环经济发展先行区和创建民族团结进步先进区的示范基地，体现“做强西宁、服务全省”的意识。“一个城市窗口”，即打造展示青海精神高地内涵的城市窗口，为全面建成小康社会提供精神动力，体现西宁的城市“软实力”。“五个排头兵”，即争当推动绿色发展、促进创新创业、深化改革开放、完善公共服务、加强法治建设的排头兵，体现西宁在全省推进“四个全面”战略布局中的地位和作用，彰显了西宁的责任担当。

同时，在完成上述目标的基础上，要努力实现人民生活水平和质量普遍提高；经济保持中高速增长；生态环境质量显著改善；公民素质和城市文明程度明显提升；各方面制度更加成熟更加定型。

据此，明确了“十三五”时期经济发展、生态文明、民生福祉、创新驱动4大类28项指标，其中预期性指标14项，约束性指标14项。在指标体系设置上，我们比国家多设置了5项预期性指标。主要是充分考虑我市发展现状与全国平均水平的差距，强调结构调整、转型升级、对外开放、追赶发展等方面的紧迫性，对我市在全省率先全面建成小康社会至关重要。

《纲要（草案）》提出的今后五年经济保持中高速增长的若干目标，主要是到2020年地区生产总值达到2010年的三倍左右，人均生产总值提前超过一万美元，这就要求今后五年的年均增速需保持在9.5%左右。一方面，从潜力上看，综合各方面测算，西宁在“十三五”时期经济潜在增长率为9%-10%，我们按照中间值来安排生产总值增长指标，既可能、也比较合适。另一方面，从支撑上看，综合对一产二产三产增长潜力和条件的分析，三次产业的增速，可以支撑9.5%左右的增长。这样安排，有利于正向引导市场预期，特别是有利于支撑全省经济增长、为我省抓好生态文明建设创造更大空间。

（三）“十三五”时期经济社会发展的重点任务

围绕实现“12315”总目标和开启“幸福西宁”建设新征程，《纲要（草案）》提出6个方面重点任务：一是强化共享发展理念，着力提升人民生活水平。二是强化创新发展理念，着力提高发展质量效益。三是强化协调发展理念，着力推动城市转型升级。四是强化绿色发展理念，着力增加生态产品供给。五是强化开放发展理念，着力打造对外开放高地。六是坚持党的领导，强化规划执行力。

各位代表，“十三五”规划凝聚着社会各方的智慧，承载着全市人民的期盼。我们要凝心聚力、接续奋斗，不断提升城市综合实力，不断提高人民生活水平，携手开启“幸福西宁”建设新征程。

三、打好实现“12315”总目标第一仗，奋力开启“幸福西宁”建设新征程

2016年是全面建成小康社会决胜阶段的开局之年，“决胜要有决心，开局重在开头”。今年工作的总体要求是：全面贯彻中央和省委关于今年经济工作的决策部署，以习近平总书记系列重要讲话为指导，按照“四个全面”战略布局和“五位一体”总体布局，牢固树立创新协调绿色开放共享的发展理念，适应经济发展新常态，把握稳中求进工作总基调，把握“131”总体要求和“12315”总目标，坚持以生态保护优先理念协调推进经济社会发展，坚持履职为民的坚定立场和价值取向，坚持稳增长、调结构、促改革、惠民生、防风险，在继续扩大有效需求的同时，着力加强供给侧结构性改革，提高供给体系质量和效率，改造提升传统比较优势，加快培育新的发展动能，增强持续增长动力，努力实现“十三五”时期经济社会发展的良好开局，开启“幸福西宁”建设新征程。

经济社会发展主要预期目标是：在优化结构、提高质量效益的基础上，地区生产总值增长9.5%左右，新增城镇就业3万人，农民转移就业32万人次，全体居民人均可支配收入增长10%左右，居民消费价格涨幅和节能减排控制在省定目标以内，空气质量优良率保持在75%以上。在完成上述目标基础上，结构性改革取得实质性进展，过剩产能和商品住房库存减少，企业成本上涨势头得到遏制，有效供给能力有所提高，财政支出结构更加合理。

做好今年的工作，重点要把握好“三个关系”：一是速度与效益质量的关系。要保持一定增长速度为调结构转方式创造有利空间，夯实稳增长的基础。同时，也不能纠结于“速度情结”和“换挡焦虑”，避免盲目追求速度造成新的结构失衡和包袱。二是传统动能提升与新动能成长的关系。既要稳住传统动力，继续加大投资力度，促进工业提质增效，提高城镇化质量，更要把握发展大逻辑，主动引领新常态，积极培育发展新动能，增强经济持续发展动力。三是稳步开局与增强后劲的关系。既要坚定信心、主动作为，保持经济平稳较快发展，又要保持定力、未雨绸缪，不为一时的经济波动而忽视经济发展中的深层次矛盾，抓住牵引全局的关键领域和环节，集中力量打好攻坚战，保持经济稳中向好、行稳致远。

抓好“六项重点”工作：

（一）全力以赴稳增长，为建设“幸福西宁”夯实物质基础。围绕在全省率先全面建成小康社会的目标，积极培育推动经济持续稳定增长的动力体系，从供需两侧发力推进结构性改革，全力以赴稳定经济增长，为全面建成小康社会打下坚实基础。

发挥投资对经济增长的关键拉动作用。投资对我市经济增长的贡献率将近60%，是需求侧当之无愧的主动力。必须坚持投资拉动不松劲，抓好市级177项重点项目建设，确保全社会固定资产投资增长10%以上。优化投资结构。调整优化投资特别是公共投资结构，投向涉及公众普遍利益和长远利益的公共产品领域和外部性比较明显的领域，更多投向科教文化、医疗卫生、健康养老、住房保障、生态环保、交通通讯等社会基础设施和公共服务领域。加大对传统产业转型升级和特色优势产业、战略性新兴产业、高新技术产业的投入，加大对科技创新、科技成果转化、新增长点领域人才培养、顶尖创新人才引进等方面的投入。狠抓重点投资。全力稳定工业投资，围绕锂电、光伏、装备制造、生物医药等新兴产业，力促签约招商和技改项目尽快落地建设。实现工业投资525亿元，增长5%以上。加快海湖批发市场、建国路、门源路等片区储备土地出让进度，积极推动绿地、碧桂园、红星美凯龙等重点项目尽快落地。完成房地产投资322亿元，增长15%以上。全面开工建设西城大街和峡口路高架、昆仑大道西延、凤凰山路、南北高速下线口等重点项目。完成城市轨道交通规划报批，年内开工建设1号线。力争基础设施投资达到318亿元，增长20%。发挥好民间投资的主力军作用，充分激发民间投资的活力和潜能。创新项目收益和分配机制，实现政府和社会资本合作（PPP）模式项目破题。强化项目保障。落实重点项目调度管理制度，强化协调衔接，主动跟进服务，加快项目土地、规划、环评审批进度，为项目建设创造有利条件。做好耕地占补平衡，将道路建设与城中村改造、棚户区改造结合起来，加快凤凰山路、湟岸巷、曹家寨等重点片区拆迁进度，完成年度土地储备和供给任务，着力解决土地制约。

发挥消费对经济增长的基础作用。围绕推动服务、信息、绿色等六大领域消费，大力发展新产品、新服务和新模式，以消费升级促进产业升级，全年社会消费品零售总额增长11%。促进旅游消费。围绕打造丝绸之路经济带特色旅游名城、中国西部区域旅游集散中心和青藏高原国际旅游服务基地目标，加大重点景区开发力度，加快建设鲁沙尔民族文化旅游产业园、丹噶尔古城二期、以西宁为起始点的自驾车营地，积极推进博物馆群5A级景区创建工作，确保成功创建全国旅游标准化示范城市。促进健康养老消费。整合各类养老服务资源，集中改扩建和新建一批农村养老服务设施，加快推进医养结合，放开养老服务市场，促进社会资本进入健康养老产业，建设以居家为基础、社区为依托、机构为补充的多层次养老服务体系，推动生活照料、康复护理、精神慰藉、紧急援助等服务全覆盖。促进住房消费。以满足新市民的住房需求为主要出发点，建立购房与租房并举、市场配置与政府保障相结合的住房制度。继续推进棚改货币化安置，加大对农牧民进城购房的财政和信贷支持力度，促进房地产业持续健康发展。开展物业服务提升年活动，通过严格监管、满意度评价、收费与服务质量挂钩、品牌企业建设等措施，全面提升物业管理水平，提高群众满意度。促进信息消费。贯彻落实“宽带中国”战略，加快网络通信基础设施建设和信息产业等新兴消费业态发展，推进物联网终端、智能终端在社会民生等领域的融合和应用，增强信息消费对经济增长的拉动作用。

着力推进供给侧结构性改革。紧抓国家结构性改革的重大历史机遇，找准五大支柱政策与西宁实际的结合点，落实好供给侧改革五大任务，力争用3到5年时间，彻底改变产业偏粗偏重偏短的状况，闯过结构调整这个转折关口，完成一场深刻变革，开创西宁发展新境界。一是去产能。按照企业主体、政府推动、市场引导、依法处置的办法，引导和倒逼落后、过剩产能退出。逐个分析产能过剩行业企业，主动出手，抢占先机，尽可能多兼并重组，少破产清算，综合施策盘活一批企业，积极争取中央专项，加快黄河再生铝等一批企业的重组力度。妥善解决人员安置、债务负担等问题，防范引发社会风险。二是降成本。着眼于降低制度性交易成本、人工成本、企业税费负担、社会保险金、要素成本，制定帮助企业降低成本政策，切实减轻企业负担。三是减库存。在消化房地产库存的同时，积极消化有色、化工等行业产成品库存，减少资金占用，有效改善企业财务状况。四是增活力。培育若干具有较强资源整合能力的骨干企业并发挥其对所处行业的整合和带动作用，注重支持成长性好的中小企业，加强对困难企业的帮扶。五是防风险。加强风险监测预警，严厉打击非法集资、金融诈骗等活动，坚决守住不发生系统性和区域性风险的底线。做好政府存量债务置换工作，推动政府融资平台市场化转型，防控政府债务风险。

发挥好财政和金融的支撑保障作用。围绕海绵城市、地下综合管廊等国家试点项目，加大专项资金、地方政府债券、专项建设基金等争取力度，确保全年专项资金总量增长20%，着力做大财力总量。实行部门专项经费零基预算，优化支出结构，继续深挖盘活存量资金，集中财力保民生、办大事。加大金融对实体经济的支持力度，促进金融资本和产业资本融合，组建金融控股公司，设立产业引导、基础设施等投资基金推动战略性新兴产业、科技创新型企业、现代服务业发展和基础设施建设。整合市属各类融资担保机构，建设市级政策性信用担保集团，全力支持中小微企业、创新创业、新型农业经营主体发展，缓解企业融资难、融资贵。拓展企业上市、企业债券、股权交易等融资渠道，扩大直接融资规模，力争全社会融资规模达到900亿元以上，增长15%。

（二）坚定不移推进产业转型升级，为建设“幸福西宁”增加实力。坚持在转型中发展，促进新动能成长和传统动能加力增效，加快培育传统产业、新兴产业等新的增长点，力促产业提质增效。

优化布局、集约发展，构建产业发展新格局。按照着力培育增长点，打造高新技术产业集聚区、多巴城市副中心、特色优势产业集群等3个增长极和拓展宁大北川高新技术产业经济带、多沙沿湟现代服务业经济带、鲁多—西塔沿线特色优势产业经济带等3条千亿元经济增长带的要求，加快编制生产力空间布局规划，建设东川、南川、城北国家级高新技术开发区、甘河和北川高新技术区、大华等6个先进制造业组团，加快形成建国路—大什字—商业巷综合商贸、海湖新区高端商务和现代金融、城北科教创新服务、南川文化旅游商贸会展、北川特色口岸综合服务、多巴文体商旅物流、鲁沙尔民族文化旅游、丹噶尔历史文化旅游等8个现代服务业板块，构建错位发展、优势互补的产业布局。

做优存量、做大增量，力促工业转型升级。西宁的跨越发展，关键在产业，核心是工业。必须更加重视工业转型升级、提速增效，力争工业增加值增长11%，新兴产业比重进一步提高。做优存量强基础。实施“百项改造提升工程”，加大技改力度，运用新技术、新工艺、新装备等提升传统产业，启动实施桥电压小上大等199项技改项目。稳定电解铝行业生产，推动高端铝材、铝镁合金等产业链延伸项目建设，提高铝水就地转化率。铁合金行业全面启动热矿炉向大型自动、密闭节能化方向改造。钢铁行业扩大高性能轴承钢、高强度钢筋等高端产品规模。水泥行业推进余热利用、富氧燃烧等技术推广应用，扩大特种水泥、高标号水泥生产规模，支持发展高标号水泥构件产品。藏毯绒纺行业加大产品研发、国外市场拓展、个性化定制等力度，提高产品档次。做大增量强实力。围绕中国制造2025战略和“互联网+”行动计划，实施“百项创新攻坚工程”项目。打造全国重要的千亿锂电基地，实施正极材料、锂电池隔膜、动力及储能电池等项目，加快构建锂电全产业链。启动单晶硅切片、光伏电池组件等项目建设，推动光伏产业上下游产业融合发展。力促汽车制造、高端数控机床、石油机械生产等项目实施，加快形成装备制造和新材料产业集群。强化培育添活力。实施“百户领军企业打造工程”，新培育规上企业不少于20户。落实“千户小微企业培育工程”和“轻工业提升工程”，全面推动88户轻工企业扩能增产，助力全市工业稳定增长。继续开展干部入企服务，落实市场主体发展倍增计划，大力支持非公企业发展、提高，增强稳增长政策措施针对性，提高企业投资信心。产城融合聚合力。支持开发区走以城聚产、以产兴城、产城联动、融合发展的路子，优先落实用地指标，大力支持园区征地拆迁、拆违打非等工作，加快园区与城市基础设施互联互通，改进交通供水供电等公共服务，为园区发展创造条件，不断提高单位土地的投资强度和产出率，推进产业园区向宜业宜居的功能性产业城区转型，提升园区辐射带动能力和对全市经济贡献率。

聚焦特色、品牌推动，打造现代农业新优势。坚持集约化、有机化、品牌化方向，以增加“菜篮子”有效供给，促进农民增收为目标，打造农业与牧业循环、规模经营与品牌效益兼得、一二三产业融合发展的高原都市现代农业升级版。全力推进三县现代农业示范区和都市休闲观光农业示范区建设，提升大通县国家级现代农业示范区，启动创建湟源县国家级现代农业示范区、湟中县省级现代农业示范区，建设西宁现代农业产业示范园。开展农药、化肥零增长行动，提升农机装备水平和设施农业建设水平，加强农田水利基本建设，改善农业生产条件，提高粮油、果蔬两个优势产业生产能力。大力发展生态畜牧业和种养结合循环农业，合理布局规模化养殖场，停止在川水地区批建高密度集中圈养式养殖场，实施养殖业出川上山，两年内完成湟水河两岸养殖企业搬迁工作。围绕打造高原绿色有机品牌，延伸农业产业链，鼓励农民合作与联合、龙头企业带动，促进种养加一体化发展，提升农畜产品精深加工水平，促进产业链增值更多的留给生产地和农民。保障财政对农业农村农民的投入，财政奖补等扶持政策向合作社、家庭农牧场等多种形式的农牧业规模主体倾斜，专项资金以股权方式投入农业经营主体，撬动更多社会资本投入农业农村，用资本杠杆助力品牌农业，着力构建多元化的资本股权纽带体系。加强乡镇村金融服务体系建设，加快农村“六类”产权确权发证，构筑县乡综合信用担保体系，大力推进“金穗服务惠万家”，解决农民贷款难、贷款贵问题。

拓宽领域、扩大规模，提升现代服务业发展水平。把加快服务业发展作为经济转型升级的战略支点，促进服务业优质高效发展，力争实现第三产业增加值增长8.5%以上。推动生产性服务业更高效。申报现代物流创新发展试点城市，狠抓丝绸之路（国际）物流城、中国（西宁）国际清真产业园等建设。加快制造业企业中营销设计等服务业的剥离，选择一批龙头企业开展制造业服务化试点，推动生产性服务业向专业化和价值链高端延伸。加大本土电商企业扶植力度，推进“电子商务进农村”。大力发展绿色金融，加快城西现代金融聚集区建设。促进生活性服务业更便民。加强商贸服务设施体系建设，打造包括物流配送、便民超市、银行网点、零售药店、家庭服务中心等在内的便捷生活服务圈。构建以批发市场为龙头，农贸市场、商场超市为主体，社区便利店和蔬菜直通车为补充的农产品流通体系。促进高端商务商贸服务业发展，加快形成海湖新区、建国路、水井巷等高端特色商贸圈，推动万达广场、丝路风情街等高端项目早日开业。

集聚要素、科技引领，增强创新驱动能力。围绕国家创新型城市创建，加快国家级和省级高新技术产业园区及青大科技园建设，组建锂电、光伏、生物医药、镁铝合金等产业技术中心和技术创新联盟，把技术创新重点从单个企业转到产业链延伸和产业集群发展上，增强关键领域、重点行业创新发展能力。鼓励大企业、科研机构向社会开放研发测试、生产装备、创新孵化等资源。建好用好西宁中关村科技成果转化基地。加快城北创新创业基地建设。支持鼓励科技中介、金融服务、中试孵化等众创服务平台建设，提升全市创新创业承载能力。加大创投、风投等基金对科技型公司的支持力度，推动各类创新要素向企业集聚。新培育认定科技型企业20家，科技研发中心10家，全年专利申请量突破1700件。坚持引进和培育相结合，启动“高端创新人才千人计划”，加快聚集高层次创新创业人才。

（三）全力推进城市转型升级，为建设“幸福西宁”构筑载体。坚持“人与自然、现代与传统、艺术与技术、建筑与环境”的和谐统一，着力抓好规划、建设、管理三大关键环节，努力把西宁建设成为“品位好、品质好、品评好”的城市。

统筹协调不失衡，构建城市发展新格局。把城市空间重构作为城市转型升级的突破口、着力点，坚持绿色为芯、双城联动、生态环抱、组团发展，加快形成以200平方公里城市绿芯森林公园为“一芯”，西宁主城区、多巴城市副中心为“双城”，南川文化旅游商务会展区、鲁沙尔民族文化旅游产业园区、城北高新技术产业创新创业聚集区、北川省级高新技术产业园区、西川新城为功能组团的“一芯双城、环状组团发展”的生态山水城市格局，打造西宁发展新版图。紧抓国家新型城镇化综合试点机遇，充分发挥县区竞相发展的积极性，突出区域协同、城乡一体，加快推进市域周边成熟地区快速城市化。着力完善市域城镇体系布局，按照小城市规划标准提高三县县城建设水平，加快重点特色镇发展，推动小城镇发展与疏解中心城区功能相结合、与特色产业发展相结合、与服务“三农”相结合。发展具有特色优势的休闲旅游、商贸物流、先进制造、民俗文化传承、科技教育等魅力小镇，带动农业现代化和农民就近城镇化。

规划引领不动摇，塑造城市特色新风貌。引入城市美学理念，补充完善涵盖全域的各类专项规划，促进“多规合一”，形成覆盖全面、层次清晰的高标准城市规划体系，切实增强城市的空间立体性、平面协调性、风貌整体性、文脉延续性，让城市有根、有魂、有魅力。编制完成《西宁市城市建筑风貌导则》，从自然风貌及其意境中寻找西宁城市特色的本底，结合西宁多民族、多宗教、多元文化交融发展的特点，在建筑风格上体现大气、质朴、雄浑的河湟文化特色，在城市布局上注重顺应自然、彰显生态山水意境。聘请国内知名设计院和大师级设计师主持重要地段城市设计和重要建筑设计，打造一批具有时代风格和鲜明特色的地标性建筑。强化城乡规划建设领导小组和专家委员会、审批委员会、监督委员会作用，切实增强规划的严肃性和权威性，杜绝随意变更。严格执行“双增双减”，从严控制高层住宅密度、数量。

夯实基础不松劲，创造优良人居新环境。进一步加大基础设施建设力度，全方位提升城市功能。全面推进“畅通西宁”三年攻坚计划，综合实施六大专项行动，建设94条276公里“外环内网”道路，加速形成“四横十五纵一环”主干路网体系，力争三年人均道路面积由7.8平方米提高到13平方米。加快建设市域“三环七射”高速公路网，在建成100公里内环高速的基础上，启动建设连接23个乡镇的市域高速中环，连通城市新空间，带动重点区域快速发展。加快城北等三个客运枢纽站场及15处公交场站建设，新（改）建公交港湾60个，新增优化公交线路26条，新增公交车辆298台。改造建设311处公共临时停车场、11380个泊位，年内投入使用，力争主城区停车难问题有所缓解。加快推进智能交通建设，健全应急指挥与处置、公众出行信息服务等软件系统，建成公安交警智能管理平台和交通运输智能管理平台，全力规范交通秩序，倡导市民文明出行，努力营造安全畅通、文明有序的交通环境。推进充电站、充电桩等新能源汽车充电设施建设。结合城市轨道交通、河道治理、道路整治等逐步推进地下综合管廊建设，今年完成泉湾路等45公里综合管廊建设，破解“马路拉链”难题。推进海绵城市建设，实现雨水自然蓄积、自然渗透、自然净化。围绕城镇构筑生态屏障、惠及群众建设园林绿地、服务通道提升形象，全力开展“美化家园”全民绿化行动，改造提升城区景观，全面开工长青园、人民公园等增绿亲水工程，建设城市滨水景观，打造水清岸绿、亲民休闲的生态河道，再建成150公里城市绿道。

各位代表，我们要下定决心，努力把这座城市的每一个细节建设得更加贴近市民，亲近群众，使之成为各族人民群众共建共享的“幸福西宁”！

强化管理不懈怠，全面提升城市新形象。深化城市管理和行政执法体制改革。健全科学合理划分市、区（县）、街（镇）三级城市管理职责，加快推进城市管理领域大部门制改革，综合设置管理执法机构。以“执法重心下移、职能下沉、权责统一、集约运行”为目标，构建以街（镇）为主体的城市综合管理体系，合理划分各级管理职权，将城区环卫保洁、市场秩序监管、违章建筑管理、违停车辆管理等职能下放至街（镇）。整合环卫作业、绿化养护、道路管护、河道管理等职能，建立市政公用一体化管理养护服务模式。调整优化执法力量，执法力量向基层倾斜，推进综合执法。巩固国家卫生城市等创建活动经验成果，深入开展“一城为总、多城联创”活动，实施“美丽夏都—清洁西宁”品牌行动和创建全国文明城市市容环境综合提升行动，提升主次大街、高速公路和国省道沿线、城市出入口、景区景点环境卫生管理水平，有效治理背街小巷、农贸市场、河道沟渠、城乡结合部等区域环境卫生问题，加强公共厕所、垃圾分类试点和中转站等环卫设施建设。不断提升市政设施、建筑立面、绿地景观建设管理水平，规范报刊亭、公交候车亭等“城市家具”设置，广泛开展“五星级文明户”创建工作，大力整治各类不文明行为，不断提高市民文明素质，形成市容环境卫生干净整洁，市民行为文明有序的城市生活环境，为建设“幸福西宁”打下坚实基础。加强智慧城市建设，大力打造政务云、公共服务云、社会服务云三大平台，促进大数据、物联网、云计算等现代信息技术与城市管理服务融合，努力为群众提供精细的城市管理和良好的公共服务。

（四）全面加强生态文明建设，为建设“幸福西宁”筑牢生态安全屏障。以生态文明统领经济社会发展全局，坚持生态保护第一，坚持绿色发展方向，建设全省生态文明先行区、循环经济发展先行区示范基地，努力走向生态文明建设新时代。

强化生态系统保护修复。划定永久基本农田、生态保护红线和城市开发边界，加大对周边森林、河湖、农田、湿地等生态主体功能区的保护。构建“一芯两屏三廊道”的城市生态屏障，启动绿芯森林公园建设，完成南绕城高速公路景观等绿化工程。抓好环城国家生态公园建设，申办中国（西宁）园林博览会，创建国家生态园林城市。加快第五、第七水源地等水源保护省级示范工程建设。全面启动农村饮水安全达标全覆盖工程。治理水土流失面积80平方公里。主动开展生态环保大检查，积极整改主要道路沿线、旅游景区周边及农村综合环境、工业园区污染、大气环境质量等社会关注度高的生态环保问题。

持续推进环境综合治理。大力实施大气污染防治工程，全面完成5000辆黄标车淘汰和119蒸吨燃煤锅炉改造任务，强力推进桥头铝电烟气脱硫、祁连山水泥粉尘治理等15个工业大气污染综合治理项目，从严控制渣土作业、道路、裸露地等扬尘污染，确保空气质量优良率保持在75%以上。持续推进湟水流域污染治理不放松，加快城市污水管网建设，推进三县、甘河东区、第一、第三污水处理厂提标改造和第六污水处理厂项目建设。加大农村环境连片整治力度，全面推进村级保洁制度，实施农村垃圾统一收集、集中处理，启动建制镇污水处理项目。

推动低碳循环绿色发展。开展“万家企业”节能降碳行动，实施东川、甘河工业园区循环化改造和甘河工业园低碳示范试点工作，在电解铝、铁合金、水泥等行业开展专项对标活动，支持重点行业企业开展清洁生产和减排技术改造，实施70个节能降耗技改项目，规上企业单位工业增加值能耗比上年下降2%以上。

（五）千方百计保障和改善民生，坚定建设“幸福西宁”的价值取向。按照人人参与、人人尽力、人人享有的要求，努力增强公共服务供给能力，不断提高民生保障水平。

大力推进脱贫攻坚。把脱贫攻坚作为头等大事和重大政治责任，坚持精准识别、摸清底数、分类施策、动态管理，今年确保80个村整村脱贫，2.6万人提前脱贫，为“十三五”时期提前实现整体稳定脱贫赢得“开门红”。全面实施“八个一批”扶贫攻坚专项行动，确保年内完成1000户、4000人搬迁安置，劳动技能培训11000人，为贫困村群众免费提供15类基本公共卫生服务项目，在37个贫困村实施高原美丽乡村建设项目。加大城市贫困群众帮扶力度，统筹抓好就业、就学、就医、住房、社保、物价等事关群众切身利益的民生工作，不断提高公共服务能力和水平。

全力以赴增收富民。全面落实国家各项增收政策，稳步增加城乡居民收入，有针对性地开展农民工技能培训，做好园区、建筑业等用工对接，巩固农民工资性收入。大力培育新兴经营主体，发展特色高效品牌农业，提高农业附加值，更加注重增加二、三产非农经营性收入，着力提高农民家庭经营性收入在农民收入中的占比。

坚持就业优先战略。实施更加积极的就业政策，以发展产业增就业、加强培训促就业、推动创业带就业，实施高校毕业生就业促进计划和创业引领计划，发挥中小企业就业主渠道作用，实现更加充分更高质量的就业。大力发展职业教育，建立完善劳动者职业技能终身培训体系。加强对灵活就业、新就业形态的支持，完善对就业和生活困难人员的就业援助。完善创业教育培训体系，提高劳动者创业能力。落实创业扶持政策，发挥创业孵化基地作用，加大创业平台建设力度，建设一批小微企业创业创新基地和创业型社区、创业型城镇。

更加关注社保托底。落实基本养老金合理调整机制，完成社保卡发行发放任务。整合临时救助、财政扶贫和社会捐助资金，建立贫困家庭高等教育全过程资助制度，保障贫困家庭子女顺利完成学业。完善临时救助制度，加大对因病致贫、因灾致贫家庭的救助力度，切实解决困难家庭燃眉之急。健全特困人员救助供养制度，确保城乡特困人员都能获得救助供养服务。制定三年计划，加快棚户区、城中村改造，加强老旧住宅小区综合整治，完善和提升小区配套设施，到2020年基本完成现有城镇棚户区、城中村改造。重视公租房配套设施建设，加快分配入住进度。

着力发展社会事业。全面启动三期教育布局结构调整工程，继续推进标准化学校建设和义务教育薄弱学校改造，加大财政对接收农民工随迁子女较多的城镇中小学校、幼儿园建设的投入力度，优先完成103个农村幼儿“走教点”改造和28个乡镇中心幼儿园建设。启动教育信息化“三通两平台”建设计划，扩大优质教育覆盖面，实施城乡贫困家庭学生15年免费教育，绝不让一个孩子输在起跑线上。推进创伤急救、眼科、妇幼保健和中西医康复等特色医疗项目建设。加快城区二级医院、村卫生室标准化改造，下沉医疗卫生资源，让每一名群众享受更加方便公平的医疗服务。全面完成国家公共文化服务体系示范区创建验收工作，启动湟中民族风情园、城西区文化创意产业园等文化产业项目。着力办好第十届中国国际民间文化艺术节、穿越丝路国际徒步节等活动。

多措并举稳控物价。加快青藏高原农副产品集散中心二期和农产品电子交易中心建设，加强与三县蔬菜基地对接，拓宽地产蔬菜销售渠道。发挥国有农产品控股公司引导作用，建成150个社区平价便民店，国有企业蔬菜供给能力达到40%。加快建设“西宁阳光物价网”，让群众及时了解全市物价信息，着力做好平价粮油等生活必需品的储备和市场投放，确保群众生活必需品价格稳定。

全面强化社会治理。坚持安全第一，健全城市应急指挥体系，完善城市生命通道系统，构建全天候、系统性、现代化的城市安全保障体系。深入推进平安西宁，实施警力配置和社会治安评价机制改革，加快构建社会治安立体防控体系。完善落实安全生产责任和管理制度，加大监管执法力度，及时排查整改隐患，坚决防止发生重特大生产安全事故。加大食品药品监管和执法力度，确保群众饮食用药安全。全力抓好国防动员和后备力量建设，促进军政军民更加团结。创新城市民族工作，促进各民族交流交往交融，建设全省创建民族团结进步先进区的示范基地。

各位代表，我们将始终坚持以人民为中心的发展思想，始终坚持履职为民的坚定立场和价值取向，确保财政对民生领域的投入保持在80%以上，把群众当成自己的亲人和依靠，继续办好十大类40项民生实事，确保各族群众共享改革发展成果，不断提高群众的获得感和幸福感！

（六）深化改革促开放，为建设“幸福西宁”提供动力源泉。突出特色抓改革，争当改革开放排头兵，先行先试、率先突破，打造改革高地。

简政放权要提高实效。严格落实“权力清单”，廓清权力边界，管住权力任性，防止乱作为；严格落实“责任清单”，明确肩上责任，管住权力懈怠，防止不作为。推进行政审批服务局优化审批流程，减少审批环节和要件，打造“模块化”行政审批模式，加强与上下级和平级部门的协同联动，建成行政审批“网上办事大厅”，保证相对集中行政审批权改革凸显实效。努力营造“亲商爱商”社会氛围和营商环境。改进县区级审批薄弱环节，解决好“放下来、接不住”的问题。放管结合，加强事中事后监管。上半年全面完成县区政府机构改革任务和公车制度改革。

大力推进重点领域改革。加强农村宅基地制度、不动产统一登记等国家重点改革任务和创建省级生态县、支农资金切块下拨等省级改革试点工作，努力创造可复制、可借鉴的经验。结合市情探索突破瓶颈的有效途径，加快城市管理体制、投融资体制等我市改革创新步伐。深化市属国有企业改革，引进优秀人才，配强领导班子，提高资本效率，建立审计监督与绩效考核挂钩的奖励约束机制。积极发展混合所有制经济，大力支持非公经济发展，提高非公经济比重。深化医药卫生体制改革，积极推进公立医院人事薪酬分配制度改革，理顺医疗服务价格，建立长效经费补偿机制，改革医保支付方式，成立医疗联合体。抓好教育综合改革，建立城乡教育一体化发展机制，推进义务教育集团化办学。

开创对外开放新局面。着力建设法治化、国际化、便利化的营商环境，打造丝绸之路经济带重要节点城市。深化与“一带一路”国家间的经贸合作，加大企业“走出去”力度，开拓国际市场。加快口岸开放体系建设，推动西宁综合保税区申建、黄金口岸国际保税购物中心等海关特殊监管区建设，力争再开通1-2条国际航线。拓展境外营销网络建设，设立一批西宁特色商品国际营销中心。实施“一带一路”重点投资项目和进口商品直营直销平台建设项目。全力办好城洽会、绿色发展论坛及夏都国际论坛，充分发挥青洽会、清食展、藏毯展、环湖赛等大型节会的招商推介平台作用，强化市级领导带头招商，产业链招商，力争实现招商引资到位资金920亿元以上。

各位代表，新形势对各级政府提出了新的更高要求。我们将切实加强政府自身建设，增强政治意识、大局意识、核心意识、看齐意识，自觉践行“三严三实”要求，认真履行职责，对人民负责，受人民监督，使人民满意。弘扬勤学善思之风。坚定正确的政治方向，深入学习贯彻习近平总书记系列重要讲话精神，不断增强落实省委“131”总体要求和市委“12315”总目标的自觉性和坚定性。加强各方面的知识学习培训，努力提升破解难题、开拓创新的本领，在建设学习型政府上见实效。严把依法行政之要。全面落实法治政府实施纲要，厘清市场、政府、社会权责边界，切实做到法无授权不可为、法定职责必须为。主动接受人大依法监督和政协民主监督，提高人大代表意见建议和政协委员提案办理质量。健全依法决策机制，重视听取社会各方面对涉及经济社会发展重大问题、重大公共利益及重大民生等的意见建议，推进行政决策科学化、民主化、法治化。加强行政监察、执法监督和审计监督，严格落实行政执法追究责任制。全面推行政务公开，依法保障人民群众的知情权、参与权、表达权和监督权，让政府更好地服务人民，让人民更好地监督政府。坚守廉洁奉公之责。坚持把纪律和规矩挺在前面，认真履行“一岗双责”，持之以恒推进政府作风建设，坚决反对和惩治腐败，坚决查处损害群众利益的行为。坚持从严从俭，刚性约束，严格控制一般性支出，坚决禁止舌尖上的浪费、车轮上的铺张和节假日期间的不正之风，切实把有限财力投入到加快发展、改善民生上。提振实干担当之气。调动各级政府主动作为的积极性，着力克服慵懒散现象，鼓励干部振奋精神愿干事，沉下身子干成事，多做事关西宁长远发展的基础性、铺垫性工作，不做急功近利的形象工程，为西宁发展奠定坚实基础；全心全意为人民服务，从群众满意的事情做起，从群众不满意的问题改起，踏踏实实把西宁人民群众的事办好，真正以实际行动赢得民心、赢得支持。

各位代表，时代赋予使命，奋斗创造未来，“十三五”蓝图已经绘就。“只要我们真心为群众谋利益，全心全意为人民服务，群众就会真心拥护、全力支持我们，西宁就会拥有不断奋勇前行的巨大力量”。全市各级政府和广大干部要牢记和践行为人民服务的根本宗旨，向党中央看齐，向习近平总书记看齐，和各族群众一道，更加紧密地团结在以习近平同志为总书记的党中央周围，在省委、省政府和市委的坚强领导下，紧紧围绕“12315”总目标，建设“幸福西宁”，改革创新，奋发进取，以“干在实处、走在前列”的精神，为全面建成小康社会、谱写“中国梦”西宁篇章而不懈奋斗！
